# Higher Dementia Risk in People With Gastroesophageal Reflux Disease: A Real-World Evidence

**DOI:** 10.3389/fnagi.2022.830729

**Published:** 2022-04-04

**Authors:** Shuo-Yan Gau, Jung-Nien Lai, Hei-Tung Yip, Meng-Che Wu, James Cheng-Chung Wei

**Affiliations:** ^1^School of Medicine, Chung Shan Medical University, Taichung, Taiwan; ^2^School of Chinese Medicine, College of Chinese Medicine, China Medical University, Taichung, Taiwan; ^3^Department of Chinese Medicine, China Medical University Hospital, Taichung, Taiwan; ^4^Management Office for Health Data, China Medical University Hospital, Taichung, Taiwan; ^5^College of Medicine, China Medical University, Taichung, Taiwan; ^6^College of Medicine, National Chung Hsing University, Taichung, Taiwan; ^7^Division of Gastroenterology, Children’s Medical Center, Taichung Veterans General Hospital, Taichung, Taiwan; ^8^Institute of Medicine, Chung Shan Medical University, Taichung, Taiwan; ^9^Department of Allergy, Immunology and Rheumatology, Chung Shan Medical University Hospital, Taichung, Taiwan; ^10^Graduate Institute of Integrated Medicine, China Medical University, Taichung, Taiwan

**Keywords:** gastroesophageal reflux disease, dementia, cohort study, epidemiology, NHIRD

## Abstract

**Background:**

Whether or not patients with gastroesophageal reflux disease (GERD) have a higher risk of developing subsequent dementia remains unknown, and no observational evidence from population-based data is available. This study was to determine whether patients with GERD have a higher future risk of developing dementia.

**Methods:**

For the period 2000–2012, datasets from the Longitudinal Health Insurance Database (LHID, subset of National Health Insurance Research Database in Taiwan) were analyzed. Definition of GERD was based on ICD-9-CM codes 530.11 and 530.81 and prescriptions for PPIs. After matching gender, age, index year, and comorbidities, each GERD patient was matched with four control patients without GERD. Future risk of dementia was evaluated, and sensitivity analysis of subgroups was conducted to clarify the potential association.

**Results:**

In the present study, 13,570 patients were included in the GERD cohort and 54,280 patients were included in the control cohort. Patients with GERD showed higher risk developing dementia than control group, with an aHR of 1.34 (95% C.I., 1.07, 1.67). In GERD patients between above 70 years old, the risk of developing dementia was higher than that of the control groups (aHR = 1.34; 95% C.I., 1.01, 1.77).

**Conclusion:**

Patients with GERD showed higher incidence of dementia, and elder patients had the highest risk of developing dementia. Clinicians should be concern of the association between GERD and dementia and should develop strategies to prevent dementia while managing patients with GERD.

## Highlights

– **Question:** Do patients with gastroesophageal reflux disease (GERD) have higher risk in developing future dementia?
– **Findings:** Patients with GERD showed higher incidence of dementia, and elder GERD patients had the highest risk of dementia occurrence.
– **Meaning:** Clinicians should be concern of the association between GERD and dementia and should develop strategies to prevent dementia while managing patients with GERD.

## Introduction

Dementia is an acquired, multiple-caused syndrome, which has a negative influence on the life quality of affected patients (Gale et al., 2018). Dementia is characterized by a range of symptoms, including impairment of memory and compromised social and occupational functions (Rossor et al., 2010). Cerebrovascular dysfunction is thought to be an etiological factor of different categories of dementia (Raz et al., 2016).

Gastroesophageal reflux disease (GERD) involves extended contact of refluxed substances with the lining of the esophagus leading to damage to the esophageal mucosa and inflammation (Altomare et al., 2013). According to epidemiological research in Taiwan, people aged between 40 to 60 years old have higher risk developing GERD; in community, the prevalence is about 25% (Hung et al., 2011). The reflux caused by GERD would possibly lead to the change in the microbiome of GI tract. Also, as a consequence of constant injury to the tract, subsequent inflammation could also cause dysregulation of cytokines in human bodies.

Previous studies reported the negative influence of reflux of gastric juice in the performance in nervous system (Dobrek et al., 2004; Wang et al., 2019). Additionally, in the pathogenesis of GERD, the inducing of cytokine production played a critical role (Altomare et al., 2013; Ustaoglu et al., 2020). Similarly, cytokine level and neuroinflammation were also crucial factors influencing the course of dementia (Calsolaro and Edison, 2016). Though GERD and dementia share common mechanisms, it remains unclear whether or not GERD is associated with subsequent onset of dementia, and large-scale studies on this issue are lacking. The orientation of this cohort study was to clarify the association between GERD and the risk of developing dementia by analyzing data from a Taiwanese population-based database.

## Materials and Methods

### Data Source

Taiwan’s compulsory single-payer National Health Insurance (NHI) program was launched in 1995. All of the NHI’s medical claims data are collected and stored in the National Health Insurance Research Database (NHIRD). In this cohort study, the Longitudinal Health Insurance Database (LHID) were utilized. Containing one million randomly chosen beneficiaries, LHID include outpatient visits, hospitalizations, and medications in its claims data. In accordance with the International Classification of Disease, Ninth. Revision, Clinical Modification (ICD-9-CM), diagnoses and prescriptions are recorded. The data were de-identified according to privacy protocols prior to release.

### Study Population

The study cohort consisted of patients with more than one outpatient record or one hospitalization for gastroesophageal reflux disease (GERD) (ICD-9-CM code 530.11, 530.81) for the period 2000–2012. As an additional criterion, we included patients with the prescription records of proton pump inhibitors (PPIs) after the diagnosis. The Taiwanese National Health Insurance Bureau requested that patients could only be prescribed PPIs treatment based on GERD diagnosis using endoscopy or a pH meter inspection for 24 h. To increase the validity of GERD diagnosis, we only enrolled patients diagnosed with endoscopy or 24-h pH monitoring who subsequently received PPIs therapy. Additionally, given that GERD and peptic ulcer disease (PUD) could exist at the same time, the coding ICD-9-CM 530.11 and 530.81 could potentially had misclassification bias, even though including patients with the prescription records of proton pump inhibitors (PPIs) after the diagnosis. In this case, we conducted a sensitivity analysis based on the population of PUD or GERD patients (ICD-9-CM 533, 530.11, 530.81) to validate the association.

**FIGURE 1:**
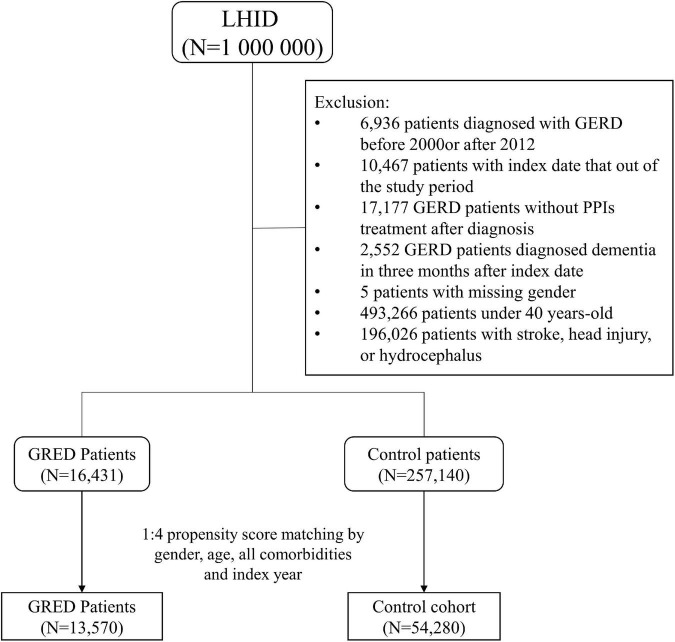
Study flow chart of patients’ selection.

Subjects having the history of GERD before the index date will not be included as the study group. The inclusion criteria were age over 40 years old and no previous dementia. Also, since dementia is cause by many risk factors, patients with a previous history of stroke (ICD-9-CM codes 430–435) head injury (ICD-9-CM codes 850–854 and 959.01), or hydrocephalus (ICD-9-CM codes 331.3, 331.4, 331.5, 741.0, and 742.3) were excluded from the study design. Each patient in the GERD cohort was matched with four control patients by propensity score. The propensity score was calculated by including gender, age, index year, and all comorbidities into a logistic regression model. The index date in the GERD group was the first diagnosis date of GERD, while in the control group the index date was a random date between 2000 and 2012. A detailed flow chart of subject recruitment is shown in Figure 1. The participants were followed up until 31 December 2013. Patients who withdrew from the NHI program or died were considered censored.

### Outcome Measurement and Covariates

The primary end-point of this study was the incidence of dementia (ICD-9-CM code 290.0-290.4, 290.8-290.9, 294, 311.0). It was defined as patients with at least two outpatient visits or one admission record. We divided age into four group: 40–49 years old, 50–59 years old, 60–69 years old, and ≥70 years old. The potential confounders were medical utilization status, related comorbidities, including hypertension (ICD-9-CM code 401-405), hyperlipidemia (ICD-9-CM code 272.0-272.4), chronic liver disease (ICD-9-CM code 571), obesity (ICD-9-CM code 278), alcohol-related diseases (ICD-9-CM code 291, 303,305.00-305.03, 571.0-571.3, 790.3, V11.3), heart failure (ICD-9-CM code 428), stroke (ICD-9-CM code 430-438), and peptic ulcer disease (ICD-9-CM code 531-533), anxiety (ICD-9-CM code 300.0, 309.2-309.4, 313.0), depression (ICD-9-code 296.2, 296.3, 296.82, 300.4, 309.0, 309.1, 309.28, 311) and PPIs medications (omeprazole, pantoprazole, lansoprazole, rabeprazole and esomeprazole).

### Statistical Analysis

Considering the large sample size of our study, using *p*-value in reporting difference in baseline characteristics might cause potential bias, for the significance might be not credible enough due to the great sample size. Thereby, we examine the difference between control and GERD cohort by standardized mean difference (SMD) to lower the influence of the bias. SMD less than 0.1 means the difference is negligible. The number of events was divided by the person-year to obtain the incidence rate. The hazard ratio and 95% confidence interval (C.I.) were estimated by the univariable Cox proportional hazard model and adjusted by the multivariable Cox proportional hazard model. The cumulative incidence curves were evaluated by the Kaplan-Meier methods and examined by the log-rank test. All statistical analysis was performed by SAS software version 9.4 (SAS Institute, Inc., Cary, NC, United States). A significance level was set as a *p*-value less than 0.05.

## Results

In total, the GERD cohort included 13,570 patients and the control cohort included 54,280 patients. As shown in Table 1, the distribution of gender and age between the two cohorts were similar after matching. Half (50%) of the participants were female and most of the participants were aged from 40 to 49 years. The mean age of the GERD patients was close to 56.2 years old. The proportion of patients developed with comorbidities were similar in the two cohorts.

**TABLE 1:** Baseline characteristics between non-GERD and GERD cohort.

	Gastroesophageal reflux disease				
	
	No (*N* = 54280)		Yes (*N* = 13570)		
		
Variables	*n*	%	*n*	%	SMD
Gender					0.014
Female	27367	50.4%	6745	49.7%	
Male	26913	49.6%	6825	50.3%	
**Age, year**					
40–49	19593	36.1%	4826	35.6%	0.011
50–59	17451	32.2%	4440	32.7%	0.012
60–69	9661	17.8%	2440	18.0%	0.005
≥70	7575	14.0%	1864	13.7%	0.006
Mean, (SD)	56.12	(11.36)	56.2	(11.34)	0.004
**Visit time (2 year before index date)**					
Median, (Q1–Q3)	28	(15–47)	38	(22–60)	0.326
**Comorbidities**					
Hypertension	20591	37.9%	5167	38.1%	0.003
Hyperlipidemia	17179	31.6%	4345	32.0%	0.008
Chronic liver disease	20377	37.5%	5280	38.9%	0.028
Obesity	605	1.1%	178	1.3%	0.028
Alcohol-related diseases	1967	3.6%	565	4.2%	0.028
Heart failure	2018	3.7%	603	4.4%	0.037
Stroke	1843	3.4%	559	4.1%	0.038
Peptic ulcer disease	34169	62.9%	8462	62.4%	0.012
Anxiety	14328	26.4%	3957	29.2%	0.062
Depression	4362	8.0%	1254	9.2%	0.043
PPIs	9707	17.9%	13570	100.0%	3.030
Omeprazole	2893	5.3%	3628	26.7%	0.610
Pantoprazole	3324	6.1%	4441	32.7%	0.714
Lansoprazole	4187	7.7%	7118	52.5%	1.117
Rabeprazole	4187	7.7%	7118	52.5%	1.117
Esomeprazole	3909	7.2%	6762	49.8%	1.071

*SMD: standardized mean difference.*

*Follow-up:*

*Non-GERD cohort = 4.79 (± 2.60).*

*ERD cohort = 4.70 (± 2.48).*

**FIGURE 2:**
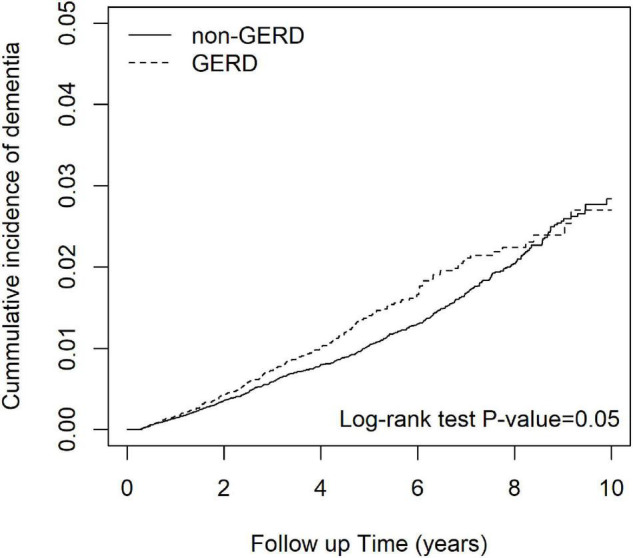
The cumulative incidence curves of patients with and without GERD.

Figure 2 shows that the cumulative incidence of dementia in the GERD cohort was significantly higher than that in the control cohort, (log-rank test: *p*-value = 0.05). The incidence rate of dementia in GERD patients was 2.73 per 1,000 person-years and that of the non-GERD patients was 2.32 per 1,000 person-year, as shown in Table 2. The adjusted hazard ratio (aHR) of developing dementia in patients with GERD was 1.34 times (95% C.I., 1.07, 1.67) that of those without GERD, for the crude hazard ratio was 1.18 (95% C.I., 1.00, 1.40). Age was certainly shown to be a risk factor for dementia, with the aHR of 16.9 in developing dementia (95% C.I., 12.71.6, 22.51). Comorbidities including hypertension, obesity, alcohol-related diseases, heart failure, stroke, peptic ulcer disease, anxiety and depression appeared to increase the risk of dementia. Generally, for all patients, uses of PPIs were associated with a decreased risk in the development of dementia, with the aHR of 0.78 (95% C.I., 0.65, 0.94).

**TABLE 2:** Cox proportional hazard model analysis.

	Dementia						
	
Variables	n	PY	IR	cHR	(95% CI)	aHR^†^	(95% CI)
Non-GERD	604	259984	2.32	1.00	(reference)	1.00	(reference)
GERD	178	65099	2.73	1.18	(1.00, 1.40)*	1.34	(1.07, 1.67)*
**Gender**							
Female	474	164261	2.89	1.00	(reference)	1.00	(reference)
Male	308	160822	1.92	0.66	(0.58, 0.77)***	0.75	(0.65, 0.87)***
**Age, year**							
40–49	62	122973	0.50	1.00	(reference)	1.00	(reference)
50–59	106	105045	1.01	2.03	(1.49, 2.78)***	1.84	(1.34, 2.52)***
60–69	161	57744	2.79	5.57	(4.15, 7.46)***	4.53	(3.35, 6.13)***
≥70	453	39321	11.5	23.8	(18.32, 31.16)***	16.9	(12.71, 22.51)***
**Comorbidities**							
**Hypertension**							
No	260	203544	1.28	1.00	(reference)	1.00	(reference)
Yes	522	121539	4.29	3.37	(2.90, 3.91)***	1.13	(0.95, 1.34)
**Hyperlipidemia**							
No	454	222351	2.04	1.00	(reference)	1.00	(reference)
Yes	328	102731	3.19	1.56	(1.35, 1.80)***	0.99	(0.85, 1.15)
**Chronic liver disease**							
No	445	195806	2.27	1.00	(reference)	1.00	(reference)
Yes	337	129277	2.61	1.11	(0.97, 1.28)	0.96	(0.83, 1.11)
**Obesity**							
No	768	321684	2.39	1.00	(reference)	1.00	(reference)
Yes	14	3399	4.12	1.76	(1.04, 2.99)*	1.84	(1.08, 3.13)*
**Alcohol-related diseases**							
No	764	313812	2.43	1.00	(reference)	1.00	(reference)
Yes	18	11271	1.60	0.67	(0.42, 1.06)	1.05	(0.65, 1.69)
**Heart failure**							
No	666	314199	2.12	1.00	(reference)	1.00	(reference)
Yes	116	10884	10.7	5.14	(4.22, 6.26)***	1.55	(1.26, 1.91)***
**Stroke**							
No	684	314041	2.18	1.00	(reference)	1.00	(reference)
Yes	98	11042	8.88	4.07	(3.29, 5.03)***	1.52	(1.22, 1.89)***
**Peptic ulcer disease**							
No	153	113219	1.35	1.00	(reference)	1.00	(reference)
Yes	629	211864	2.97	2.13	(1.79, 2.54)***	1.39	(1.16, 1.67)***
**Stroke**							
No	467	230285	2.03	1.00	(reference)	1.00	(reference)
Yes	315	94797	3.32	1.59	(1.37, 1.83)***	1.10	(0.94, 1.29)
**Depression**							
No	653	297838	2.19	1.00	(reference)	1.00	(reference)
Yes	129	27245	4.73	2.13	(1.76, 2.57)***	1.67	(1.37, 2.05)***
**PPIS**							
No	455	205113	2.22	1.00	(reference)	1.00	(reference)
Yes	327	119970	2.73	1.19	(1.04, 1.38)*	0.78	(0.65, 0.94)*

*: p-value < 0.05; **: p-value < 0.01; ***: p-value < 0.001.

*n: number of events; PY: person-years; IR: incidence rate (per 1,000 person-years).*

*cHR: crude hazard ratio; aHR: adjusted hazard ratio.*

*^†^: adjusted by sex, age, visit frequency, comorbidities and PPIS.*

**FIGURE 3:**
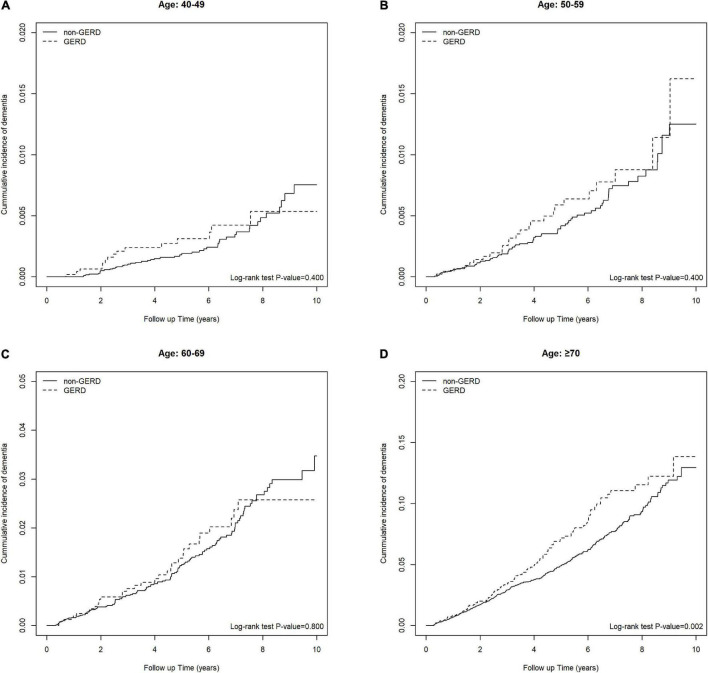
**(A)** Keplan Meier plot of the cumulative incidence of developing future dementia in people between 40–49 years old. **(B)** Keplan Meier plot of the cumulative incidence of developing future dementia in people between 50–59 years old. **(C)** Keplan Meier plot of the cumulative incidence of developing future dementia in people between 60–69 years old. **(D)** Keplan Meier plot of the cumulative incidence of developing future dementia in people >70 years old.

Table 3 shows the association of GERD and dementia stratified by gender, age groups, and comorbidities. GERD increased the risk of dementia in females by 1.40-fold (95% C.I., 1.05, 1.86). In the subgroup of patients below 70 years old, the influence of GERD to the development of dementia was not statistically significant. Figures 3A–D showed the cumulative incidence of dementia of each age subgroups. The GERD cohort showed significantly higher risk comparing with the control cohort in GERD patients aged 70 years above (aHR = 1.34, 95% C.I., 1.01,1.77). For patients without comorbidities, the relationship between GERD and dementia was significant. PPIs users with GERD showed an increased risk of dementia comparing with PPIs users without GERD, with an aHR of 1.32 (95% CI, 1.06, 1.66). Additionally, the sensitivity model based on population of PUD or GERD was presented in Table 4. PUD did not change the trend the association, for people with PUD or GERD also showed an increased risk for dementia, with an aHR of 1.50 (95% CI, 1.22, 1.85).

**TABLE 3:** The risk of dementia in different stratification level.

	Non-GERD			GERD						
		
Variables	n	PY	IR	n	PY	IR	cHR	(95% CI)	aHR^†^	(95% CI)
**Gender**										
Female	364	131577	2.77	110	32684	3.37	1.23	(0.99, 1.52)	1.40	(1.05, 1.86)*
Male	240	128407	1.87	68	32415	2.10	1.13	(0.86, 1.47)	1.28	(0.90, 1.82)
**Age, year**										
40–49	47	98423	0.48	15	24550	0.61	1.28	(0.72, 2.29)	1.08	(0.49, 2.38)
50–59	81	83433	0.97	25	21612	1.16	1.23	(0.78, 1.93)	1.64	(0.84, 3.17)
60–69	128	46332	2.76	33	11412	2.89	1.06	(0.72, 1.55)	1.29	(0.77, 2.16)
≥70	348	31796	10.9	105	7525	13.95	1.28	(1.03, 1.60)*	1.34	(1.01, 1.77)*
**Comorbidities**										
**Hypertension**										
No	205	162702	1.26	55	40843	1.3466	1.07	(0.79, 1.44)	1.40	(0.92, 2.14)
Yes	399	97282	4.1015	123	24256	5.0708	1.25	(1.02, 1.53)*	1.33	(1.03, 1.73)*
**Hyperlipidemia**										
No	342	177868	1.92	112	44483	2.52	1.31	(1.06, 1.62)*	1.44	(1.08, 1.93)*
Yes	262	82116	3.19	66	20616	3.20	1.02	(0.78, 1.34)	1.20	(0.85, 1.70)
**Chronic liver disease**										
No	326	157208	2.07	119	38598	3.08	1.49	(1.21, 1.84)***	1.58	(1.18, 2.13)**
Yes	278	102776	2.70	59	26501	2.23	0.83	(0.63, 1.10)	1.00	(0.7, 1.41)
**Obesity**										
No	591	257439	2.30	177	64246	2.76	1.21	(1.02, 1.43)*	1.36	(1.09, 1.70)**
Yes	13	2545	5.11	1	853	1.17	0.23	(0.03, 1.73)	0.36	(0.03, 3.95)
**Alcohol-related diseases**										
No	589	251138	2.35	175	62675	2.79	1.20	(1.01, 1.42)*	1.38	(1.1, 1.73)**
Yes	15	8847	1.70	3	2424	1.24	0.74	(0.21, 2.56)	0.44	(0.1, 1.88)
**Heart failure**										
No	519	251532	2.06	147	62667	2.35	1.14	(0.95, 1.38)	1.29	(1.01, 1.65)*
Yes	85	8452	10.06	31	2432	12.75	1.26	(0.83, 1.90)	1.53	(0.9, 2.58)
**Stroke**										
No	528	251560	2.10	156	62480	2.50	1.20	(1.00, 1.43)*	1.42	(1.12, 1.80)**
Yes	76	8424	9.02	22	2619	8.40	0.92	(0.57, 1.47)	0.91	(0.50, 1.66)
**Peptic ulcer disease**										
No	112	90740	1.23	41	22479	1.82	1.49	(1.04, 2.13)*	1.12	(0.64, 1.94)
Yes	492	169244	2.91	137	42620	3.21	1.11	(0.92, 1.34)	1.34	(1.05, 1.70)*
**Anxiety**										
No	354	185797	1.91	113	44489	2.54	1.34	(1.08, 1.66)**	1.48	(1.11, 1.97)**
Yes	250	74187	3.37	65	20610	3.15	0.94	(0.72, 1.24)	1.13	(0.80, 1.59)
**Depression**										
No	508	238909	2.13	145	58930	2.46	1.16	(0.97, 1.40)	1.33	(1.04, 1.69)*
Yes	96	21075	4.56	33	6169	5.35	1.18	(0.79, 1.75)	1.38	(0.81, 2.36)
**PPIS**										
No	455	205113	2.22	0	65099	0.00				
Yes	149	54871.04	2.7155	178	65098.87	2.7343	1.06	(0.85, 1.32)	1.32	(1.06, 1.66)*

*: p-value < 0.05; **: p-value < 0.01; ***: p-value < 0.001.

*n: number of events; PY: person-years; IR: incidence rate (per 1,000 person-years).*

*cHR: crude hazard ratio; aHR: adjusted hazard ratio.*

*^†^: adjusted by sex, age, visit frequency, comorbidities and PPIS.*

**TABLE 4:** Sensitivity Analysis: Population with peptic ulcer disease (PUD) or GERD.

	Dementia						
	
Variables	n	PY	IR	cHR	(95% CI)	aHR^†^	(95% CI)
Non-PUD + GERD	112	90740	1.23	1.00	(reference)	1.00	(reference)
PUD + GERD	670	234343	2.86	2.25	(1.85, 2.75)***	1.50	(1.22, 1.85)***
**Gender**							
Female	474	164261	2.89	1.00	(reference)	1.00	(reference)
Male	308	160822	1.92	0.66	(0.58, 0.77)***	0.75	(0.65, 0.87)***
**Age, year**							
40–49	62	122973	0.50	1.00	(reference)	1.00	(reference)
50–59	106	105045	1.01	2.03	(1.49, 2.78)***	1.84	(1.34, 2.52)***
60–69	161	57744	2.79	5.57	(4.15, 7.46)***	4.51	(3.33, 6.11)***
≥ 70	453	39321	11.5	23.8	(18.32, 31.16)***	16.8	(12.64, 22.38)***
**Comorbidities**							
**Hypertension**							
No	260	203544	1.28	1.00	(reference)	1.00	(reference)
Yes	522	121539	4.29	3.37	(2.9, 3.91)***	1.13	(0.95, 1.33)
**Hyperlipidemia**							
No	454	222351	2.04	1.00	(reference)	1.00	(reference)
Yes	328	102731	3.19	1.56	(1.35, 1.8)***	0.99	(0.85, 1.15)
**Chronic liver disease**							
No	445	195806	2.27	1.00	(reference)	1.00	(reference)
Yes	337	129277	2.61	1.11	(0.97, 1.28)	0.96	(0.83, 1.11)
**Obesity**							
No	768	321684	2.39	1.00	(reference)	1.00	(reference)
Yes	14	3399	4.12	1.76	(1.04, 2.99)*	1.82	(1.07, 3.10)*
**Alcohol-related diseases**							
No	764	313812	2.43	1.00	(reference)	1.00	(reference)
Yes	18	11271	1.60	0.67	(0.42, 1.06)	1.06	(0.66, 1.70)
**Heart failure**							
No	666	314199	2.12	1.00	(reference)	1.00	(reference)
Yes	116	10884	10.7	5.14	(4.22, 6.26)***	1.53	(1.25, 1.89)***
**Stroke**							
No	684	314041	2.18	1.00	(reference)	1.00	(reference)
Yes	98	11042	8.88	4.07	(3.29, 5.03)***	1.53	(1.23, 1.90)***
**Stroke**							
No	467	230285	2.03	1.00	(reference)	1.00	(reference)
Yes	315	94797	3.32	1.59	(1.37, 1.83)***	1.10	(0.94, 1.28)
**Depression**							
No	653	297838	2.19	1.00	(reference)	1.00	(reference)
Yes	129	27245	4.73	2.13	(1.76, 2.57)***	1.68	(1.37, 2.05)***
**PPIS**							
No	455	205113	2.22	1.00	(reference)	1.00	(reference)
Yes	327	119970	2.73	1.19	(1.04, 1.38)*	0.86	(0.74, 1.00)

*: p-value < 0.05; **: p-value < 0.01; ***: p-value < 0.001.

*n, number of events; PY, person-years; IR, incidence rate (per 1,000 person-years);*

*cHR, crude hazard ratio; aHR, adjusted hazard ratio. cDDDs: average cumulative defined daily dose per follow up year.*

*^†^: adjusted by sex, age, visit frequency, comorbidities and PPIS.*

Tables 5, 6 showed the influence of cumulative defined daily dose (cDDD) of PPIs and incidence of dementia among GERD cohort and control cohort. For people without GERD, increased cDDD did not have a consistent influence on the trend of development of future dementia. However, for people with GERD, dose dependent effect was observed. For GERD patients with higher cDDD of PPIs, the risk of future dementia significantly increased. GERD patients with >325 cDDD per follow up year have a 3.93-fold risk comparing with those with <70 cDDD per follow up year.

**TABLE 5:** The effect of PPIS on control patients.

	Demetria						
	
	n	PY	IR	cHR	(95% CI)	aHR	(95% CI)
**PPIs, cDDDs**							
non-user	455	205113	2.22	1.00	(reference)	1.00	(reference)
≤ 28	86	37617	2.29	0.95	(0.76, 1.20)	0.70	(0.55, 0.88)**
>28	63	17254	3.65	1.60	(1.23, 2.09)***	1.04	(0.79, 1.35)
p for trend					0.009		<0.001
**PPIs, cDDDs**							
non-user	455	205113	2.22	1.00	(reference)	1.00	(reference)
≤ 84	128	50914	2.51	1.06	(0.87, 1.29)	0.76	(0.62, 0.93)**
>84	21	3957	5.31	2.45	(1.58, 3.79)***	1.36	(0.87, 2.11)
p for trend					0.2247		<0.001

*p-value < 0.05; **: p-value < 0.01; ***: p-value < 0.001.

*n, number of events; PY, person-years; IR, incidence rate (per 1,000 person-years);*

*cHR, crude hazard ratio; aHR, adjusted hazard ratio. cDDDs: average cumulative defined daily dose per follow up year.*

*^†^: adjusted by sex, age visit frequency and all comorbidities.*

**TABLE 6:** The effect of PPIS on GERD patients.

	Dementia						
	
	n	PY	IR	cHR	(95% CI)	aHR	(95% CI)
**PPIs,cDDDs**							
<10	23	11660	1.97	1.00	(reference)	1.00	(reference)
10–28	29	17971	1.61	0.82	(0.47, 1.42)	0.89	(0.52, 1.55)
28–66	39	18790	2.08	1.08	(0.64, 1.81)	1.15	(0.68, 1.92)
>66	87	16677	5.22	2.84	(1.79, 4.50)***	2.54	(1.59, 4.06)***
p for trend					<0.001		<0.001
**PPIs,cDDDs**							
<70	93	49573	1.88	1.00	(reference)	1.00	(reference)
71–160	44	10702	4.11	2.31	(1.61, 3.31)***	2.16	(1.5, 3.11)***
161–325	24	3471	6.91	4.01	(2.56, 6.29)***	3.08	(1.94, 4.88)***
>325	17	1353	12.57	7.33	(4.36, 12.31)***	3.93	(2.32, 6.68)***
p for trend					<0.001		<0.001

*: p-value < 0.05; **: p-value < 0.01; ***: p-value < 0.001.

*n, number of events; PY, person-years; IR, incidence rate (per 1,000 person-years);*

*cHR, crude hazard ratio; aHR, adjusted hazard ratio. cDDDs: average cumulative defined daily dose per follow up year.*

*^†^: adjusted by sex, age, visit frequency and all comorbidities.*

## Discussion

The present longitudinal retrospective cohort study is the first population-based research to evaluate the risk of developing dementia in patients with GERD. Our study indicates that patients with GERD showed a higher risk of subsequently developing dementia, with an adjusted hazard ratio of 1.34 (95% C.I., 1.07, 1.67). Comparing with other age subgroups, GERD patients aged above 70 years old showed higher risk of developing dementia.

No epidemiological studies have discussed the relationship between GERD and dementia to date. However, the two diseases share a number of possible mechanisms. With prolonged exposure to gastric juice and stimulation of esophageal epithelial cells, GERD elevates chemokine level and attracts immune cells, leading to epithelial damage (Altomare et al., 2013). GERD patients were observed to have a higher IL-6 level which affects esophageal contractility (Rieder et al., 2007). Accordingly, a previous meta-analysis study indicated that inflammation markers like Interleukin-6 and C-reactive protein are related to an enhanced risk of dementia (Darweesh et al., 2018). In the present study, we considered the use of PPIs as one of the confounding factors in developing dementia. It was still ambiguous whether or not PPIs were hazardous to the development of dementia, for both results had been reported in previous literatures (Booker et al., 2016; Goldstein et al., 2017; Chen et al., 2020). In our study, the result suggested that the use of PPIs was not related to an increased risk in developing dementia (aHR = 0.78, 95% C.I., 0.65, 0.94); however, for patients with GERD, the risk of developing dementia still remained significant with a 1.34-fold risk, supporting our assumption of the association between GERD and subsequent dementia occurrence.

Aging is viewed as a major risk factor of dementia (Niccoli et al., 2017), and our results support this concept, with a significantly increased aHR in the older subgroups (aHR = 16.9, 95% C.I., 12.716, 22.51). The association between GERD and dementia was numerically higher in elder GERD patients with the age above 70 years old, with a 1.34-fold risk in developing dementia. We suppose GERD could be a potential prodrome of dementia, for the process of neurodegeneration and aging could possibly lead to GI motility dysfunction (Soenen et al., 2016). Another possible assumption was regarding the deterioration of gut microbiota stability caused by aging (Cryan et al., 2019). Esophageal diseases such as GERD lead to an imbalance of specific bacteria species in the gut and play a role in microbiota dysbiosis (Di Pilato et al., 2016). The possible interaction between gut microbiota and brain included affecting autonomic nervous activity, enteroendocrine signaling and activating innate immune system (Cryan et al., 2019). Dysbiotic microbiota seems to be linked to the occurrence of neurodegenerative diseases, and the related immune activation influences the gut-brain axis and causes neuroinflammation in the enteric nervous system (Quigley, 2017). Metabolites, or Substances produced by gut microbiota, for instance, indole molecules, fatty acids and neurotransmitters, were discussed in previous studies and reported to have influence the development of dementia (Colombo et al., 2021; Pappolla et al., 2021; Qian et al., 2021). The results of our study suggest that immune-related reactions caused by a dysbiotic microbiome in GERD patients might have relation to higher risk of later-onset dementia development in susceptible elder adults above 70 years old. Aging-related immune dysregulation, leading to a chronic inflammatory state and aging-related dysbiosis might be the causes. We suspected that GERD may worsen pre-existing dysbiosis and immune dysregulation in elder patients and thus increase the risk of dementia. Furthermore, unstable mental status might also explain the correlation. Stress played a role in the development of both GERD and dementia (Greenberg et al., 2014; Tack and Pandolfino, 2018). A cross-sectional study revealed that GERD is associated with a significant risk of developing depression and anxiety; nevertheless, in both early life and late life, depression is considered a critical risk factor and prodrome of dementia (Bennett and Thomas, 2014; Choi et al., 2018). Accordingly, we speculate that the association of GERD with higher subsequent occurrence of dementia might be due, at least in part, to GERD-related mental health disorders. Moreover, respiratory consequences associated with reflux events in the distal esophagus, such as coughing and wheezing, might occur under GERD, whether or not reflux reaches the airway (Houghton et al., 2016). Previous studies indicated that impaired respiratory function and lung diseases were associated increased risk of dementia (Yaffe et al., 2011; Lutsey et al., 2019). Further studies should focus on mechanisms that are shared by GERD and dementia to clarify the correlations.

This study had a number of strengths. To the best of our knowledge, this investigation is the first to provide an observational evaluation of the association between GERD and the following incidence of dementia. In this study, all data were obtained from the NHIRD, so recall and selection bias were minimized. Nonetheless, some limitations must be addressed. First, risk factors related to dementia, such as genetic information, serological data and lifestyle, were not considered in the analysis of this study, for those information were not available in the NHIRD. Second, the use of ICD-9 codes might not be precise enough to serve as accurate definitions of diseases. In NHIRD, the classification between subgroups of dementias such as late-onset Alzheimer’s Disease (LOAD) and Alzheimer’s Disease and Related Disorders (ADRD) were not precise enough. Hence misclassification bias could exist. However, we tried our best to address misclassification bias by utilizing definitions of exposure and outcome that has been validated by previous studies (Chang et al., 2016; Chen et al., 2016; Ling et al., 2021; Ma et al., 2021). The definition of GERD in this study included not only ICD-9 diagnosis records, but also prescription of PPIs. In Taiwan, PPIs can only be prescribed after confirming a diagnosis of GERD through endoscopy or a 24-h pH meter inspection; hence, it is likely the definition to be accurate. Moreover, the applied definition was also utilized by previous studies Though this could possibly improve the validity of GERD definition, in such case, the study design might not be applied in other countries. Third, medical surveillance bias could potentially influence the result. Given that people with GERD could have higher tendency to visit medical institutions, they could be more possible to be diagnosed as dementia. To address the issue of medical surveillance bias, medical utilization status has been added as one of the adjusted covariates (Gau et al., 2021a,b). Fourth, the influence of over-the-counter (OTC) drugs using could cause potential biases. Nevertheless, the information or OTC drugs were not enrolled in NHIRD, hence unable to be analyzed in this study. Because of the observational nature of the study designation, though several methods were applied to prevent possible confounders, unmeasurable biases could still remain.

This robust population-based cohort study provides epidemiological result on the incidence of dementia in people with GERD, and the data proved that GERD had association with a higher risk of future dementia. Physicians should be aware of this risk and develop strategies to prevent dementia in patients with chronic GERD. Future studies are needed to identify the precise mechanism underlying the association between GERD and dementia and to clarify the potential role of aging in the pathogenesis.

## Data Availability Statement

Datasets from the Longitudinal Health Insurance Database (LHID) 2000 were retrieved in this retrospective cohort study, and the data are available from the Taiwan National Health Insurance (NHI) Bureau. The data are not publicly available because of legal restrictions regarding the “Personal Information Protection Act” in Taiwan. Requests to access these datasets should be directed to Taiwan National Health Insurance (NHI) Bureau (https://dep.mohw.gov.tw/DOS/cp-2516-3591-113.html).

## Ethics Statement

The studies involving human participants were reviewed and approved by Institutional Review Board of China Medical University, with the IRB permit number CMUH104-REC2-115(CR-5). Written informed consent for participation was not required for this study in accordance with the national legislation and the institutional requirements.

## Author Contributions

S-YG, J-NL, M-CW, and JW: study conception and design. J-NL, H-TY and JW: data acquisition. S-YG, H-TY, M-CW, and JW: data analysis and demonstration and original draft preparation. All authors involved in drafting or revising the article and approved of the submitted version.

## Conflict of Interest

The authors declare that the research was conducted in the absence of any commercial or financial relationships that could be construed as a potential conflict of interest.

## Publisher’s Note

All claims expressed in this article are solely those of the authors and do not necessarily represent those of their affiliated organizations, or those of the publisher, the editors and the reviewers. Any product that may be evaluated in this article, or claim that may be made by its manufacturer, is not guaranteed or endorsed by the publisher.

## Abbreviations

GERD: gastroesophageal reflux disease
LHID: Longitudinal Health Insurance Database
aHR: adjusted Hazard Ratio
NHIRD: National Health Insurance Research Database
PPIs: Proton Pump Inhibitors
ICD-9-CM, International Classification of Disease: Ninth. Revision Clinical Modification
95% C.I.: 95% confidence interval
SMD: standardized mean difference
OTC drugs: over-the-counter drugs.
